# 1-[3-(2-Benz­yloxy-6-hy­droxy-4-methyl­phen­yl)-5-[3,5-bis­(tri­fluoro­meth­yl)phen­yl]-4,5-di­hydro-1*H*-pyrazol-1-yl]propane-1-one

**DOI:** 10.1107/S160053681301180X

**Published:** 2013-05-04

**Authors:** U. H. Patel, S. A. Gandhi, V. M. Barot, N. V. S. Varma

**Affiliations:** a Department of Physics, Sardar Patel University, Vallabh Vidyanagar, Gujarat 388 120, India; b P. G. Center in Chemistry, Smt. S. M. Panchal Science College, Talod, Gujarat 383 215, India

## Abstract

In the title compound, C_28_H_24_F_6_N_2_O_3_, the mean plane of the central pyrazoline ring forms dihedral angles of 2.08 (9) and 69.02 (16)° with the 2-benz­yloxy-6-hy­droxy-4-methyl­phenyl and 3,5-bis­(tri­fluoro­meth­yl)phenyl rings, respectively. The dihedral angle between the mean planes of the pyrazoline and 3,5-bis­(tri­fluoro­meth­yl)phenyl rings is 68.97 (9)°. An intra­molecular O—H⋯N hydrogen bond is observed, which forms an *S*(6) graph-set motif. In the crystal, pairs of weak C—H⋯F halogen inter­actions link the mol­ecules into inversion dimers while molecular chains along [100] are formed by C—H⋯O contacts.

## Related literature

For pharmacalogical and anti­cancer properties of pyrazoline derivatives, see: Smith *et al.* (2001 ▶). For graph-set motifs, see: Bernstein *et al.*, (1995 ▶). For related structures, see: Patel *et al.* (2007 ▶, 2012 ▶).
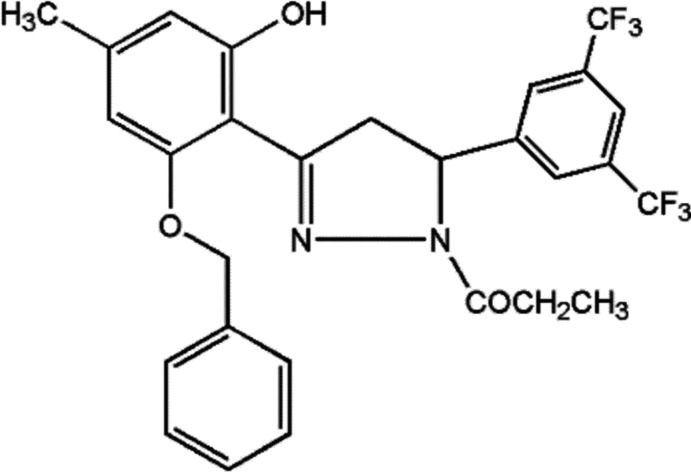


## Experimental

### 

#### Crystal data

C_28_H_24_F_6_N_2_O_3_
*M* *_r_* = 550.49
Monoclinic, 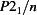

*a* = 4.8822 (2) Å
*b* = 23.4752 (9) Å
*c* = 22.4311 (9) Å
β = 91.494 (2)°
*V* = 2569.97 (18) Å^3^
*Z* = 4
Mo *K*α radiation
μ = 0.12 mm^−1^
*T* = 273 K
0.54 × 0.34 × 0.10 mm

#### Data collection

Bruker Kappa APEXII CCD diffractometer
Absorption correction: multi-scan (*SADABS*; Bruker, 2008 ▶) *T*_min_ = 0.951, *T*_max_ = 0.988
19610 measured reflections
4514 independent reflections
2491 reflections with *I* > 2σ(*I*)
*R*_int_ = 0.075

#### Refinement

*R*[*F* ^2^ > 2σ(*F* ^2^)] = 0.070
*wR*(*F* ^2^) = 0.246
*S* = 1.00
4514 reflections
352 parameters
H-atom parameters constrained
Δρ_max_ = 0.56 e Å^−3^
Δρ_min_ = −0.41 e Å^−3^

### 

Data collection: *APEX2* (Bruker, 2008 ▶ ); cell refinement: *SAINT* (Bruker, 2008 ▶ ); data reduction: *SAINT*; program(s) used to solve structure: *SHELXS97* (Sheldrick, 2008 ▶); program(s) used to refine structure: *SHELXL97* (Sheldrick, 2008 ▶); molecular graphics: *PLATON* (Spek, 2009 ▶); software used to prepare material for publication: *publCIF* (Westrip, 2010 ▶).

## Supplementary Material

Crystal structure: contains datablock(s) global, I. DOI: 10.1107/S160053681301180X/jj2162sup1.cif

Structure factors: contains datablock(s) I. DOI: 10.1107/S160053681301180X/jj2162Isup2.hkl

Supplementary material file. DOI: 10.1107/S160053681301180X/jj2162Isup3.cml

Additional supplementary materials:  crystallographic information; 3D view; checkCIF report

## Figures and Tables

**Table 1:** Hydrogen-bond geometry (Å, °)

*D*—H⋯*A*	*D*—H	H⋯*A*	*D*⋯*A*	*D*—H⋯*A*
O16—H16⋯N2	0.82	1.84	2.568 (3)	147
C31—H31⋯O9^i^	0.93	2.67	3.490 (4)	148
C21—H21⋯F37^ii^	0.93	2.72	3.488 (6)	140
C29—H29⋯F33^iii^	0.93	2.64	3.490 (5)	152

Symmetry codes: (i) 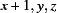; (ii) 
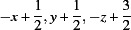
; (iii) 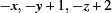.

**Fig. 1.**
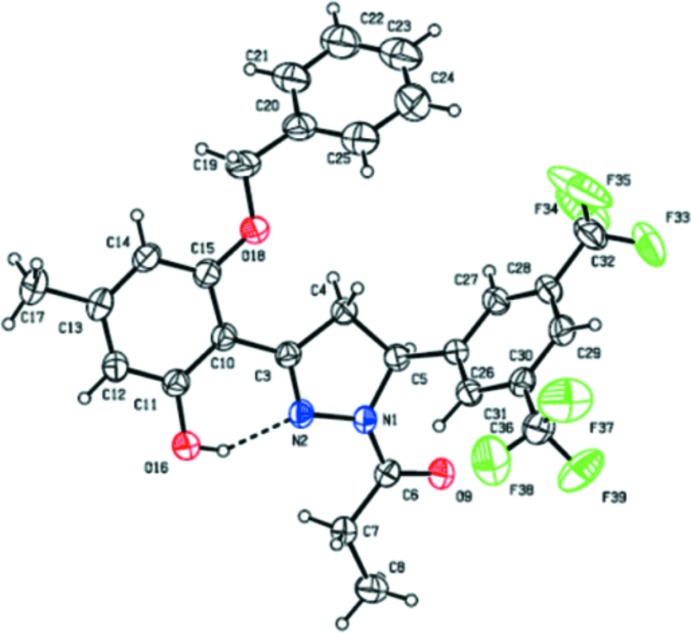
Molecular structure of the title compound,showing the atom labeling scheme with 50% probability displacement ellipsoids. The dashed line represents an O—H···N intramolecular hydrogen bond which forms an S1,1(6) graph set motif.

**Fig. 2.**
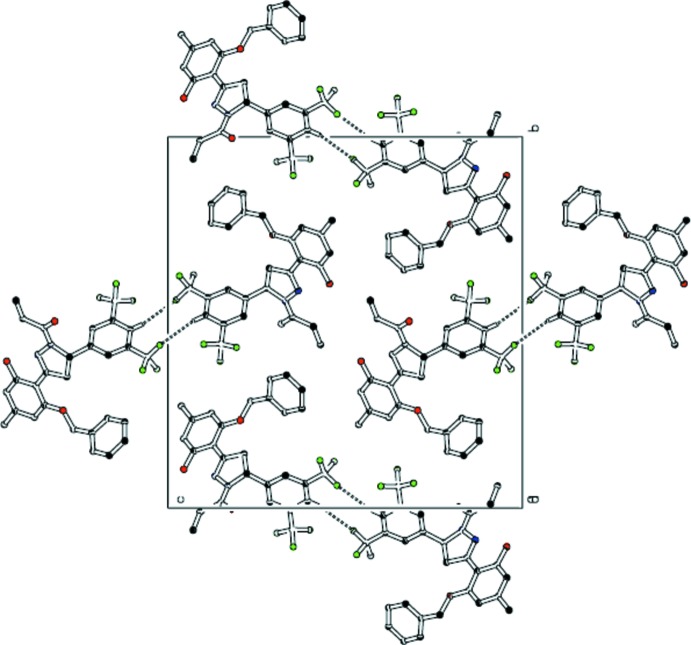
Molecular packing diagram the title compound. Dashed lines indicate weak C—H···F halogen intermolecular interactions which are displayed as inversion dimers. H atoms not involved with these weak intermolecular interactions have been deleted for clarity.

## supplementary crystallographic information

### Comment

Pyrazoline derivatives, prominent nitrogen containing heterocyclic compounds, play an important role in medicinal chemistry. These derivatives are found to possess antidepressant, antioxidant, anti- inflammatory (Smith *et al.*, 2001), anticonvulsant, antimicrobial, antiviral, monoamine oxidase (MAO-A and MAO-B) inhibitor, and anticancer activity. Our on-going research is focused on the synthesis and crystal structures of related pyrazoline derivatives of these types of heterocyclic compounds (Patel *et al.* 2007, Patel *et al.* 2012). We report here the synthesis and crystal structure of the title compound, C_28_H_24_N_2_O_3_F_6_, (I).

In (I), propionaldehyde, 3-benzyloxy-5-methyl-phenol and 1,3-bis-trifluro methyl benzene are bonded to N1, C3 and C5 of the pyrazoline ring, respectively. The dihedral angles between the mean planes of the pyrazoline ring and the 3-benzyloxy-5-methyl-phenol (C10—C15) and 1,3-bis-trifluro methyl benzene rings (C26—C31) are 2.03 (15)° and 69.02 (16)° respectively. An intra-molecular O16—H16···N2 hydrogen bond (Fig.1) forms an S1,1(6) graph set motif configuration (Bernstein *et al.*, 1995). The mean plane of the propionaldehyde group (C6—C8/O9) is inclined by 12.56 (13)° to the meam plane of the pyrazoline ring. Weak C—H···F Halogen intermolecular interactions are observed that form inversion dimers (Fig. 2).

### Experimental

1-[2-benzyloxy-6-hydroxy-4-methyl phenyl]-3-(3, 5-bis (trifluoromethyl) phenyl)prop-2-en-1-one (5 g m, 0.01 mole), hydrazine hydrate (0.70 g m, 0.014 mole) and butanoic acid (20 ml) were heated to 115–120 °C for 4 h. The resulting solution was concentrated and allowed to cool and then poured into ice. The resulting solid was filtered, washed with water, dried and rystallized from methanol as pale yellow needles.

### Refinement

All of the H atoms were placed in their calculated positions and then refined using the riding model with C—H lengths of 0.93Å, 0.98Å (CH) or 0.96Å (CH_3_) and O—H lengths of 0.82Å. The isotropic displacement parameters for these atoms were set to 1.19 to 1.20 (CH, CH_2_), 1.50 (CH_3_) or 1.49 (OH) times *U*_eq_ of the parent atom.

### Crystal data

**Table:**

C_28_H_24_F_6_N_2_O_3_	*Z* = 4
*M**_r_* = 550.49	*F*(000) = 1136
Monoclinic, *P*2_1_/*n*	*D*_x_ = 1.423 Mg m^−^^3^
Hall symbol: -P 2yn	Mo *K*α radiation, λ = 0.71073 Å
*a* = 4.8822 (2) Å	θ = 2.9–27.7°
*b* = 23.4752 (9) Å	µ = 0.12 mm^−^^1^
*c* = 22.4311 (9) Å	*T* = 273 K
β = 91.494 (2)°	Needle, white
*V* = 2569.97 (18) Å^3^	0.54 × 0.34 × 0.10 mm

### Data collection

**Table:**

Bruker Kappa APEXII CCD diffractometer	4514 independent reflections
Radiation source: sealed X-ray tube	2491 reflections with *I* > 2σ(*I*)
Graphite monochromator	*R*_int_ = 0.075
CCD scans	θ_max_ = 25.0°, θ_min_ = 2.9°
Absorption correction: multi-scan (*SADABS*; Bruker, 2008)	*h* = −5→5
*T*_min_ = 0.951, *T*_max_ = 0.988	*k* = −27→27
19610 measured reflections	*l* = −26→26

### Refinement

**Table:**

Refinement on *F*^2^	Primary atom site location: structure-invariant direct methods
Least-squares matrix: full	Secondary atom site location: difference Fourier map
*R*[*F*^2^ > 2σ(*F*^2^)] = 0.070	Hydrogen site location: inferred from neighbouring sites
*wR*(*F*^2^) = 0.246	H-atom parameters constrained
*S* = 1.00	*w* = 1/[σ^2^(*F*_o_^2^) + (0.1524*P*)^2^] where *P* = (*F*_o_^2^ + 2*F*_c_^2^)/3
4514 reflections	(Δ/σ)_max_ < 0.001
352 parameters	Δρ_max_ = 0.56 e Å^−^^3^
0 restraints	Δρ_min_ = −0.41 e Å^−^^3^

### Special details

**Table:**

Experimental. IR (cm^-1^): 2929 (C—H str. (*asym*) alkyl), 1458 (C—H def (*asym*)alkyl), 1387(C—H def (*sym*) alkyl), 3072 (C—H str.arom.), 1593. (C=Cstr. arom.), 1128 (C—H i.p.def arom.), 847 (C—H o.o.p.def.arom.), 1272 (C—O—C (*sym*) ether), 1066 (C—O—C (*asym*) ether), 3380(OH_str_. phenol). 1659 (N—CO&not; CH_2_CH_3_), 1379 (N-COCH_2_CH_3_ def., pyrazoline), 1588 (C=N str., pyrazoline), 2863 (C—H ring (str.), pyrazoline), 682 (C—H def. of CH_2_, pyrazoline), 1197 (C—N str., pyrazoline), 1117 (C—F str.).1H NMR (CDCl_3_) δp.p.m.: 0.96 (trip, 3H, J=7.3 &7.2 Hz), 1.70 (Qur, 2H),2.31(s,3*H*), 3.36 (dd, 1H, J=15.3 & 3.5 Hz), 3.83 (dd, 1H, J=11.7 & 7.2 Hz), 5.02 (s, 2H), 5.32 (trip, 1H, J= 3.1 & 8.08 Hz), 6.33 (S, 1H), 6.52 (S,1H), 7.16–7.23(m, 8H), 11.24 (s, 1H).^13^C NMR (CDCl_3_) δp.p.m.: 21.98(C-1, CH_3_), 70.91 (C-2,CH_2_ C6 H5), 159.37(C-3,Pyrazoline), 46.49 (C-4,Pyrazoline), 57.92 (C-5, Pyrazoline), 170.03(C-6,CO CH_2_ CH_3_), 36.15 (C-7,CO CH_2_ CH_3_), 13.83 (C-8, CO CH_2_ CH_3_), 124.44(C-9, CF_3_), 124.44 (C-10, CF_3_).
Geometry. All e.s.d.'s (except the e.s.d. in the dihedral angle between two l.s. planes) are estimated using the full covariance matrix. The cell e.s.d.'s are taken into account individually in the estimation of e.s.d.'s in distances, angles and torsion angles; correlations between e.s.d.'s in cell parameters are only used when they are defined by crystal symmetry. An approximate (isotropic) treatment of cell e.s.d.'s is used for estimating e.s.d.'s involving l.s. planes.
Refinement. Refinement of *F*^2^ against ALL reflections. The weighted *R*-factor *wR* and goodness of fit *S* are based on *F*^2^, conventional *R*-factors *R* are based on *F*, with *F* set to zero for negative *F*^2^. The threshold expression of *F*^2^ > σ(*F*^2^) is used only for calculating *R*-factors(gt) *etc*. and is not relevant to the choice of reflections for refinement. *R*-factors based on *F*^2^ are statistically about twice as large as those based on *F*, and *R*- factors based on ALL data will be even larger.

### Fractional atomic coordinates and isotropic or equivalent isotropic displacement parameters (Å^2^)

**Table:**

	*x*	*y*	*z*	*U*_iso_*/*U*_eq_	
N1	0.1214 (5)	0.55900 (10)	0.66924 (11)	0.0539 (7)	
N2	0.3088 (5)	0.58603 (11)	0.63303 (10)	0.0543 (7)	
C3	0.3815 (6)	0.63423 (12)	0.65664 (13)	0.0496 (8)	
C4	0.2432 (7)	0.64580 (13)	0.71404 (14)	0.0579 (8)	
H4A	0.135	0.6804	0.7115	0.069*	
H4B	0.3757	0.6492	0.7468	0.069*	
C5	0.0589 (6)	0.59313 (13)	0.72178 (13)	0.0515 (8)	
H5	−0.1338	0.6048	0.7195	0.062*	
C6	−0.0355 (7)	0.51471 (13)	0.64937 (14)	0.0543 (8)	
C7	0.0403 (7)	0.48937 (15)	0.59035 (15)	0.0658 (9)	
H7A	0.2281	0.476	0.5933	0.079*	
H7B	0.0315	0.519	0.5602	0.079*	
C8	−0.1418 (8)	0.44063 (16)	0.57049 (18)	0.0830 (12)	
H8A	−0.0827	0.4265	0.5327	0.124*	
H8B	−0.3277	0.4537	0.5665	0.124*	
H8C	−0.1307	0.4106	0.5995	0.124*	
O9	−0.2171 (5)	0.49645 (10)	0.68000 (10)	0.0699 (7)	
C10	0.5820 (6)	0.67000 (13)	0.62685 (13)	0.0532 (8)	
C11	0.6938 (7)	0.65374 (13)	0.57238 (14)	0.0571 (8)	
C12	0.8825 (7)	0.68729 (15)	0.54402 (15)	0.0654 (9)	
H12	0.9499	0.6755	0.5076	0.079*	
C13	0.9730 (7)	0.73805 (15)	0.56867 (17)	0.0640 (9)	
C14	0.8683 (7)	0.75504 (14)	0.62281 (16)	0.0657 (10)	
H14	0.9278	0.7889	0.6402	0.079*	
C15	0.6766 (7)	0.72210 (14)	0.65103 (15)	0.0605 (9)	
O16	0.6203 (5)	0.60455 (10)	0.54461 (10)	0.0770 (8)	
H16	0.5078	0.5877	0.5646	0.115*	
C17	1.1739 (8)	0.77503 (17)	0.53729 (18)	0.0844 (12)	
H17A	1.2123	0.8083	0.5609	0.127*	
H17B	1.0976	0.7863	0.4992	0.127*	
H17C	1.3404	0.7542	0.5317	0.127*	
O18	0.5648 (6)	0.73696 (10)	0.70381 (11)	0.0824 (8)	
C19	0.6523 (9)	0.78764 (16)	0.73329 (19)	0.0842 (12)	
H19A	0.6385	0.8197	0.7061	0.101*	
H19B	0.842	0.7839	0.7467	0.101*	
C20	0.4748 (8)	0.79714 (18)	0.78508 (18)	0.0758 (11)	
C21	0.3242 (9)	0.8464 (2)	0.7888 (2)	0.0907 (13)	
H21	0.3401	0.8733	0.7586	0.109*	
C22	0.1532 (11)	0.8579 (3)	0.8341 (3)	0.1063 (15)	
H22	0.0572	0.8921	0.8352	0.128*	
C23	0.1262 (10)	0.8189 (3)	0.8770 (3)	0.1106 (17)	
H23	0.0065	0.8262	0.9077	0.133*	
C24	0.2689 (13)	0.7684 (3)	0.8775 (2)	0.1151 (17)	
H24	0.2502	0.742	0.908	0.138*	
C25	0.4458 (10)	0.7582 (2)	0.8295 (2)	0.0996 (14)	
H25	0.5438	0.7242	0.8283	0.12*	
C26	0.1172 (6)	0.56381 (12)	0.78105 (13)	0.0485 (8)	
C27	0.0104 (7)	0.58758 (14)	0.83107 (14)	0.0611 (9)	
H27	−0.1031	0.6192	0.827	0.073*	
C28	0.0657 (8)	0.56612 (15)	0.88733 (15)	0.0690 (10)	
C29	0.2329 (8)	0.51930 (16)	0.89386 (15)	0.0690 (10)	
H29	0.2723	0.5044	0.9315	0.083*	
C30	0.3422 (7)	0.49453 (13)	0.84349 (15)	0.0603 (9)	
C31	0.2878 (6)	0.51677 (13)	0.78742 (14)	0.0546 (8)	
H31	0.365	0.5003	0.7541	0.065*	
C32	−0.0504 (12)	0.5936 (2)	0.94053 (18)	0.0946 (14)	
C36	0.5136 (10)	0.44223 (17)	0.8505 (2)	0.0807 (12)	
F37	0.3614 (6)	0.39586 (11)	0.85326 (17)	0.1375 (12)	
F38	0.6768 (7)	0.43269 (13)	0.80719 (16)	0.1480 (14)	
F39	0.6615 (8)	0.44101 (13)	0.89967 (16)	0.1546 (14)	
F33	−0.1600 (12)	0.56096 (17)	0.97657 (17)	0.219 (3)	
F34	0.1149 (8)	0.6254 (3)	0.9680 (2)	0.235 (3)	
F35	−0.2551 (10)	0.6284 (2)	0.92844 (15)	0.190 (2)	

### Atomic displacement parameters (Å^2^)

**Table:**

	*U*^11^	*U*^22^	*U*^33^	*U*^12^	*U*^13^	*U*^23^
N1	0.0651 (17)	0.0526 (16)	0.0442 (15)	−0.0098 (13)	0.0082 (12)	0.0046 (12)
N2	0.0614 (16)	0.0565 (17)	0.0448 (15)	−0.0068 (13)	0.0004 (12)	0.0073 (12)
C3	0.0554 (18)	0.0452 (18)	0.0480 (18)	0.0006 (14)	−0.0038 (14)	0.0049 (14)
C4	0.071 (2)	0.0496 (18)	0.054 (2)	0.0050 (15)	0.0087 (16)	0.0039 (15)
C5	0.0540 (18)	0.0507 (18)	0.0499 (18)	0.0048 (14)	0.0050 (14)	0.0034 (14)
C6	0.0599 (19)	0.0476 (18)	0.055 (2)	−0.0019 (15)	0.0010 (16)	0.0059 (15)
C7	0.074 (2)	0.064 (2)	0.059 (2)	−0.0114 (17)	0.0156 (17)	−0.0002 (16)
C8	0.091 (3)	0.076 (3)	0.083 (3)	−0.017 (2)	0.015 (2)	−0.023 (2)
O9	0.0737 (15)	0.0703 (16)	0.0666 (16)	−0.0161 (12)	0.0206 (12)	0.0007 (12)
C10	0.0591 (19)	0.0504 (18)	0.0498 (19)	−0.0019 (15)	0.0004 (15)	0.0066 (14)
C11	0.067 (2)	0.0492 (18)	0.055 (2)	−0.0003 (16)	−0.0012 (16)	0.0047 (16)
C12	0.073 (2)	0.065 (2)	0.059 (2)	−0.0020 (18)	0.0139 (17)	0.0091 (17)
C13	0.062 (2)	0.059 (2)	0.071 (2)	−0.0037 (17)	0.0012 (17)	0.0181 (18)
C14	0.080 (2)	0.0501 (19)	0.067 (2)	−0.0122 (17)	0.0000 (19)	0.0046 (16)
C15	0.066 (2)	0.059 (2)	0.056 (2)	−0.0091 (16)	−0.0026 (16)	0.0033 (16)
O16	0.0990 (19)	0.0673 (16)	0.0659 (16)	−0.0194 (13)	0.0254 (13)	−0.0101 (12)
C17	0.084 (3)	0.077 (3)	0.093 (3)	−0.017 (2)	0.012 (2)	0.022 (2)
O18	0.108 (2)	0.0729 (17)	0.0669 (17)	−0.0386 (14)	0.0217 (14)	−0.0174 (13)
C19	0.094 (3)	0.067 (2)	0.092 (3)	−0.022 (2)	0.011 (2)	−0.020 (2)
C20	0.082 (3)	0.070 (3)	0.075 (3)	−0.015 (2)	−0.001 (2)	−0.019 (2)
C21	0.095 (3)	0.089 (3)	0.089 (3)	−0.005 (3)	0.003 (2)	−0.026 (2)
C22	0.112 (4)	0.104 (4)	0.103 (4)	−0.005 (3)	0.006 (3)	−0.019 (3)
C23	0.100 (4)	0.123 (5)	0.109 (4)	−0.007 (3)	0.007 (3)	−0.048 (4)
C24	0.147 (5)	0.117 (4)	0.081 (4)	−0.023 (4)	0.004 (3)	−0.006 (3)
C25	0.112 (4)	0.094 (3)	0.093 (4)	−0.004 (3)	0.000 (3)	−0.011 (3)
C26	0.0526 (18)	0.0464 (17)	0.0467 (18)	−0.0022 (13)	0.0056 (14)	0.0002 (13)
C27	0.072 (2)	0.0548 (19)	0.057 (2)	0.0073 (16)	0.0118 (17)	0.0005 (16)
C28	0.095 (3)	0.063 (2)	0.050 (2)	0.003 (2)	0.0152 (18)	−0.0002 (17)
C29	0.090 (3)	0.069 (2)	0.048 (2)	−0.011 (2)	0.0017 (18)	0.0079 (17)
C30	0.071 (2)	0.0503 (19)	0.060 (2)	−0.0009 (16)	0.0009 (17)	0.0112 (16)
C31	0.0609 (19)	0.0533 (19)	0.0497 (19)	0.0025 (15)	0.0049 (15)	0.0012 (15)
C32	0.141 (4)	0.098 (3)	0.046 (2)	0.014 (3)	0.010 (3)	−0.008 (2)
C36	0.099 (3)	0.065 (3)	0.078 (3)	0.011 (2)	−0.009 (3)	0.018 (2)
F37	0.136 (2)	0.0594 (16)	0.216 (3)	0.0071 (15)	−0.006 (2)	0.0306 (18)
F38	0.167 (3)	0.128 (2)	0.152 (3)	0.088 (2)	0.065 (2)	0.053 (2)
F39	0.187 (3)	0.125 (2)	0.148 (3)	0.057 (2)	−0.078 (2)	0.0105 (19)
F33	0.400 (7)	0.148 (3)	0.119 (3)	0.008 (4)	0.160 (4)	−0.001 (2)
F34	0.156 (3)	0.362 (7)	0.188 (4)	−0.068 (4)	0.061 (3)	−0.206 (5)
F35	0.242 (4)	0.229 (4)	0.102 (2)	0.119 (4)	0.033 (3)	−0.038 (2)

### Geometric parameters (Å, º)

**Table:**

N1—C6	1.360 (4)	C17—H17C	0.96
N1—N2	1.392 (3)	O18—C19	1.421 (4)
N1—C5	1.464 (4)	C19—C20	1.484 (5)
N2—C3	1.295 (4)	C19—H19A	0.97
C3—C10	1.464 (4)	C19—H19B	0.97
C3—C4	1.495 (4)	C20—C25	1.362 (6)
C4—C5	1.541 (4)	C20—C21	1.374 (6)
C4—H4A	0.97	C21—C22	1.359 (6)
C4—H4B	0.97	C21—H21	0.93
C5—C26	1.517 (4)	C22—C23	1.338 (7)
C5—H5	0.98	C22—H22	0.93
C6—O9	1.214 (4)	C23—C24	1.376 (8)
C6—C7	1.506 (5)	C23—H23	0.93
C7—C8	1.509 (5)	C24—C25	1.418 (7)
C7—H7A	0.97	C24—H24	0.93
C7—H7B	0.97	C25—H25	0.93
C8—H8A	0.96	C26—C27	1.368 (4)
C8—H8B	0.96	C26—C31	1.388 (4)
C8—H8C	0.96	C27—C28	1.379 (5)
C10—C11	1.404 (4)	C27—H27	0.93
C10—C15	1.411 (4)	C28—C29	1.374 (5)
C11—O16	1.356 (4)	C28—C32	1.482 (5)
C11—C12	1.380 (5)	C29—C30	1.390 (5)
C12—C13	1.381 (5)	C29—H29	0.93
C12—H12	0.93	C30—C31	1.381 (4)
C13—C14	1.389 (5)	C30—C36	1.492 (5)
C13—C17	1.499 (5)	C31—H31	0.93
C14—C15	1.380 (5)	C32—F33	1.244 (6)
C14—H14	0.93	C32—F34	1.251 (6)
C15—O18	1.362 (4)	C32—F35	1.315 (6)
O16—H16	0.82	C36—F38	1.291 (5)
C17—H17A	0.96	C36—F39	1.303 (5)
C17—H17B	0.96	C36—F37	1.320 (5)
			
C6—N1—N2	122.0 (3)	H17A—C17—H17C	109.5
C6—N1—C5	123.7 (3)	H17B—C17—H17C	109.5
N2—N1—C5	112.0 (2)	C15—O18—C19	119.7 (3)
C3—N2—N1	109.7 (2)	O18—C19—C20	108.4 (3)
N2—C3—C10	119.6 (3)	O18—C19—H19A	110
N2—C3—C4	112.7 (3)	C20—C19—H19A	110
C10—C3—C4	127.7 (3)	O18—C19—H19B	110
C3—C4—C5	103.3 (2)	C20—C19—H19B	110
C3—C4—H4A	111.1	H19A—C19—H19B	108.4
C5—C4—H4A	111.1	C25—C20—C21	117.1 (4)
C3—C4—H4B	111.1	C25—C20—C19	123.0 (4)
C5—C4—H4B	111.1	C21—C20—C19	119.9 (4)
H4A—C4—H4B	109.1	C22—C21—C20	123.5 (5)
N1—C5—C26	114.8 (2)	C22—C21—H21	118.2
N1—C5—C4	102.3 (2)	C20—C21—H21	118.2
C26—C5—C4	111.5 (2)	C23—C22—C21	118.5 (5)
N1—C5—H5	109.3	C23—C22—H22	120.8
C26—C5—H5	109.3	C21—C22—H22	120.8
C4—C5—H5	109.3	C22—C23—C24	122.4 (5)
O9—C6—N1	119.9 (3)	C22—C23—H23	118.8
O9—C6—C7	123.9 (3)	C24—C23—H23	118.8
N1—C6—C7	116.2 (3)	C23—C24—C25	117.3 (5)
C6—C7—C8	113.8 (3)	C23—C24—H24	121.4
C6—C7—H7A	108.8	C25—C24—H24	121.4
C8—C7—H7A	108.8	C20—C25—C24	121.2 (5)
C6—C7—H7B	108.8	C20—C25—H25	119.4
C8—C7—H7B	108.8	C24—C25—H25	119.4
H7A—C7—H7B	107.7	C27—C26—C31	118.7 (3)
C7—C8—H8A	109.5	C27—C26—C5	117.8 (3)
C7—C8—H8B	109.5	C31—C26—C5	123.3 (3)
H8A—C8—H8B	109.5	C26—C27—C28	122.1 (3)
C7—C8—H8C	109.5	C26—C27—H27	118.9
H8A—C8—H8C	109.5	C28—C27—H27	118.9
H8B—C8—H8C	109.5	C29—C28—C27	119.4 (3)
C11—C10—C15	116.1 (3)	C29—C28—C32	120.0 (3)
C11—C10—C3	121.4 (3)	C27—C28—C32	120.6 (4)
C15—C10—C3	122.5 (3)	C28—C29—C30	119.2 (3)
O16—C11—C12	116.5 (3)	C28—C29—H29	120.4
O16—C11—C10	121.8 (3)	C30—C29—H29	120.4
C12—C11—C10	121.7 (3)	C31—C30—C29	120.9 (3)
C11—C12—C13	121.2 (3)	C31—C30—C36	120.1 (3)
C11—C12—H12	119.4	C29—C30—C36	119.0 (3)
C13—C12—H12	119.4	C30—C31—C26	119.7 (3)
C12—C13—C14	118.5 (3)	C30—C31—H31	120.2
C12—C13—C17	121.2 (4)	C26—C31—H31	120.2
C14—C13—C17	120.3 (3)	F33—C32—F34	109.3 (5)
C15—C14—C13	120.6 (3)	F33—C32—F35	100.3 (5)
C15—C14—H14	119.7	F34—C32—F35	101.9 (5)
C13—C14—H14	119.7	F33—C32—C28	115.8 (4)
O18—C15—C14	123.2 (3)	F34—C32—C28	113.7 (5)
O18—C15—C10	114.9 (3)	F35—C32—C28	114.2 (4)
C14—C15—C10	121.9 (3)	F38—C36—F39	107.1 (4)
C11—O16—H16	109.5	F38—C36—F37	104.6 (4)
C13—C17—H17A	109.5	F39—C36—F37	104.0 (3)
C13—C17—H17B	109.5	F38—C36—C30	114.8 (3)
H17A—C17—H17B	109.5	F39—C36—C30	113.8 (4)
C13—C17—H17C	109.5	F37—C36—C30	111.6 (4)
			
N1—C6—C7—C8	−179.7 (3)	C15—O18—C19—C20	173.2 (3)
C14—C15—O18—C19	−2.2 (5)	O18—C19—C20—C21	−120.8 (4)

### Hydrogen-bond geometry (Å, º)

**Table:**

*D*—H···*A*	*D*—H	H···*A*	*D*···*A*	*D*—H···*A*
O16—H16···N2	0.82	1.84	2.568 (3)	147
C31—H31···O9^i^	0.93	2.67	3.490 (4)	148
C21—H21···F37^ii^	0.93	2.72	3.488 (6)	140
C29—H29···F33^iii^	0.93	2.64	3.490 (5)	152

Symmetry codes: (i) *x*+1, *y*, *z*; (ii) −*x*+1/2, *y*+1/2, −*z*+3/2; (iii) −*x*, −*y*+1, −*z*+2.
